# Recommendations for reviewing manuscripts of the article types “project report” and “how to” for the GMS Journal for Medical Education

**DOI:** 10.3205/zma001691

**Published:** 2024-09-16

**Authors:** Katrin Schüttpelz-Brauns, Angelika Homberg, Marianne Giesler, Achim Schneider, Pia Gadewoltz, Martin Boeker, Andreas Möltner, Jan Matthes

**Affiliations:** 1 Medical Faculty Mannheim at Heidelberg University, Department of Medical Education Research of the Division of Studies and Teaching Development, Mannheim, Germany; 2 Freiburg/Br., Germany; 3 University of Ulm, Faculty of Medicine, Department of Studies and Teaching, Ulm, Germany; 4 Bielefeld University,Faculty of Medicine OWL, Department of Studies and Teaching, Bielefeld, Germany; 5 Technical University of Munich, School of Medicine and Health, Klinikum rechts der Isar, Chair of Medical Informatics, Institute for Artificial Intelligence and Informatics in Medicine, Munich, Germany; 6 Heidelberg University, Competence Center for Examinations in Medicine, Heidelberg, Germany; 7 University of Cologne, Faculty of Medicine, Center for Pharmacology, Cologne, Germany

**Keywords:** medical education, project report, how to, peer review

## Abstract

**Objective:**

This position paper of the Committee on Methodology in Educational Research sets out the criteria for the acceptance, revision, or rejection of manuscripts of the article types *project report* and *how to* in the GMS Journal for Medical Education, as well as outlining the development of these criteria.

**Methods:**

In a workshop with writers, reviewers, and editors, we formulated and discussed common core elements for articles. We did this by consulting the journal’s editorial board on the basis of guidelines for authors and reviewers from other journals and by using examples of articles considered less or more successful. From this, we derived specific aspects to be addressed and rejection criteria for the respective article types.

**Results:**

We have identified the target group, relevance, justification, and implication as the common core elements for both article types. We have also derived specific aspects to be addressed and rejection criteria from these core elements for each article type.

**Conclusion:**

A manuscript lacking core elements will be rejected. If aspects are not described sufficiently or are not clearly comprehensible, the manuscript must be revised.

## Zusammenfassung

**Zielsetzung:**

Dieses Positionspapier des Ausschusses für Methodik der Ausbildungsforschung beschreibt Bewertungskriterien zu Annahme, Überarbeitung oder Ablehnung von Manuskripten der Artikeltypen *Projektbericht* und *Gewusst wie* beim GMS Journal for Medical Education und deren Entwicklung.

**Methodik:**

Im Rahmen eines Workshops mit schreibenden, begutachtenden und herausgebenden Personen wurden in Rücksprache mit der Schriftleitung auf Grundlage von Richtlinien für Autor*innen und Reviewer*innen anderer Zeitschriften sowie anhand von gelungenen und weniger gelungenen Beispielartikeln gemeinsame Kernelemente für Artikel formuliert und diskutiert. Daraus wurden konkrete zu adressierende Aspekte und Ablehnungskriterien für die jeweiligen Artikeltypen abgeleitet.

**Ergebnisse:**

Als gemeinsame Kernelemente für beide Artikeltypen wurden Zielgruppe, Relevanz, Rechtfertigung sowie Implikation identifiziert. Aus diesen Kernelementen abgeleitete spezifische zu adressierende Aspekte und Ablehnungskriterien werden für den jeweiligen Artikeltyp vorgestellt.

**Schlussfolgerung:**

Das Fehlen von Kernelementen in einem Manuskript kann Grund für dessen Ablehnung sein. Überarbeitungsbedarf ist gegeben, wenn zu adressierende Aspekte nicht ausreichend oder nicht gut nachvollziehbar beschrieben werden.

## 1. Background

Medical education research journals publish different types of articles. In addition to original articles, there are, for example, reports on innovations that present novel solutions to problems and challenges in education [1], [2], [3], as well as specific types of articles that deal with the practical implementation of certain procedures, such as the introduction of new teaching and assessment formats.

The article types are named differently in the different journals, e.g. publications on innovations are published under the following headings:

- Innovation Reports in Academic Medicine [4],
- Discursive Articles in Anatomical Science Education [5],
- Project Report in the GMS Journal for Medical Education [6],
- Innovations in Medical Education in the Journal of General Internal Medicine [7],
- Educational Innovation in the Journal of Graduate Medical Education [8] or
- Really Good Stuff in Medical Education [9].

The following sections are available for article types that deal with the practical implementation of teaching, learning, and assessment formats:

- The Clinical Teacher’s Toolbox in The Clinical Teacher [10],
- How to in the GMS Journal for Medical Education [6] or
- Twelve Tips in the Medical Teacher [11].

Research papers must follow a structure based on the IMRaD scheme: introduction, methods, results, and discussion. This same structure is used for structuring the acceptance and rejection criteria, as exemplified by the GMS Journal for Medical Education (GMS J Med Educ) [12]. These criteria are missing for the article types just described. Colbert and Getz (2021) identified a wide range of key features in innovation reports in their review [13]. However, only two of the twelve characteristics were found to be congruent. These were the description of the problem and the description of the implementation of the innovation. All other characteristics varied across the journals and sometimes even within a single journal. It is evident that specifications are required for the *innovation report* article type, and that corresponding evaluation criteria must be derived. A meeting of the Editorial Board of GMS J Med Educ also revealed the need for a specification of the article types *project report* (corresponds to the article type *innovation report*) and *how to*, as well as the need for clear evaluation criteria for reviewers. As a result, the *Committee on Methodology in Educational Research* of the *DACH Association for Medical Education* (GMA) undertook the task of developing appropriate criteria.

These criteria are intended to ensure that

- authors can write their articles in a more targeted manner, e.g., to reduce the probability of a revision being needed or even the rejection of the submission due to content or qualitative deficiencies.
- Reviewers are supported in their work to ensure they arrive independently at reviews that are as congruent as possible and not fundamentally contradictory.
- Editors are supported to make more transparent and consistent decisions about manuscripts.
- The overall quality of publications of these two article types is demonstrably improved.
- Readers should be able to find articles of good quality and also associate the magazine with a certain profile or expect a certain range of contributions.

This position paper sets out the criteria for accepting, revising, or rejecting manuscripts of the article types *project report* and *how to* in the GMS J Med Educ. The first step is to identify (1) the general core elements for all article types. Core elements are superordinate characteristics that must be present. Based on these core elements, (2) necessary aspects to be addressed can then be derived, and (3) rejection criteria can be formulated. This is necessary because there is very little information about article type *how to* as opposed to article type *project report*. 

## 2. Methodology

### 2.1. Participants 

Eight experts from the *Committee on Methodology in Educational Research* plus one editor-in-chief of the GMS J Med Educ were available. All participants were experienced authors, seven of them were also experienced reviewers, and one individual was a member of the editorial board of GMS J Med Educ.

### 2.2. Material 

In addition to the expertise of the individuals involved, the development process was based on the guidelines for authors of relevant journals in the field of medical education in which comparable article types are published. Furthermore, if available, the corresponding review criteria of these journals were taken into account. This primarily concerned the journals Anatomical Science Education [5], Medical Education [9], The Clinical Teacher [10], and Medical Teacher [11], as their guidelines contain detailed information on the review of and requirements for these article types.

In addition, *project reports* in GMS J Med Educ were selected as exemplars of successful and less successful reporting practices. The sole published *how to* in GMS J Med Educ was consulted. 

### 2.3. Procedure

#### 2.3.1. Preparation phase

In the initial phase, the importance of the two article formats, particularly in the context of the GMS J Med Educ journal, was determined in accordance with the *guidelines for authors* and following consultation with the editorial board. In the second step, comparable article types from other journals in the field of medical education were identified. Corresponding guidelines for authors and reviewers were then sought, and, where available, respective review criteria were compiled. In the third step, criteria were compiled with reference to the materials described in 2.2, which, from the participants’ point of view, can assist reviewers in accepting, revising, or rejecting a manuscript.

#### 2.3.2. Workshop

In January 2023, the experts engaged in a one-day on-site work phase, during which they discussed, selected, rephrased as necessary, and categorized the criteria based on prepared examples of *project reports*. The diverse viewpoints of the participants, including those of writers, reviewers, and members of the editorial board, were explicitly incorporated into the process. The categories were designated, and the criteria were assigned via a moderated group discussion until all participants reached a consensus on the outcome. The authors identified common core elements from the aforementioned consented criteria, which can be applied to all article types. This was done in order to subsequently derive criteria for the relatively new article type *how t*o for which only a limited number of examples were available.

## 3. Results

### 3.1. Common core elements 

*Target group, relevance, justification*, and *implications* were identified as common core elements for *project report *and *how to*, which also apply to original articles but must be defined according to the respective article type (see table 1 (Tab. 1)). The* target group* determines the importance of the other core elements. For the target group of researchers, the objective is to gain knowledge, with the research methodology serving as a means of justifying the results and their significance. For practicing educators, the practical applicability of solutions to problems is typically of greater consequence than theoretical considerations. The target groups of research and/or practice also vary across journals. This must also be considered when reviewing manuscripts. The *relevance *of an article is contingent upon the expectations of the target audience with respect to the specific type of article in question. The authors should ensure that the added value for the respective target group is clearly evident, regardless of whether the objective is to gain knowledge or to solve a practical problem. The core element of *justification *is to ensure the reliability of outcomes or the soundness of proposed solutions. The core element *implications* focuses more on the theoretical implications, i.e., the contribution to knowledge in the subject area and/or the practical usefulness of the solutions presented, depending on the type of article.

The core elements can be used to specify review criteria. A need for revision arises when the core elements are unclear or inadequately described in the manuscript being reviewed. If core elements are completely missing, the article should be rejected. 

### 3.2. Aspects to be addressed and rejection criteria for the GMS J Med Educ article type project report 

The aim of *project reports* is to present a problem in medical education and to develop (innovative) solutions. A *project report* should present a common problem, possible solutions, and, of course, the project itself and its contribution to solving the problem. The argumentation of the problem, the selection of the solution approaches, the project objective, and the implementation must be presented in a comprehensible way. The manuscript must be revised if it lacks a recognizable, consistent structure or if the content is not described in a comprehensible and coherent manner. Aspects to be addressed and the derived rejection criteria are presented in table 2 (Tab. 2). 

### 3.3. Aspects to be addressed and rejection criteria for the GMS J Med article type how to 

The aim of the *how to *article type in the GMS J Med Educ is to present practical tips on a topic or conceptual considerations for undergraduate, graduate, and postgraduate education. Again, the core elements will need to be addressed. In addition to justifying the need for the article and defining the target group, the implementation instructions should be comprehensible, feasible, and at a similar level of abstraction. Aspects to be addressed and rejection criteria are listed in table 3 (Tab. 3). 

## 4. Discussion

With respect to writing and reviewing educational research articles, common core elements have been identified that need to be appropriately defined for each article type. Based on this, aspects to be addressed (by the authors) and evaluation criteria to be considered (by the reviewers) were agreed upon for the *project report* and *how to* article types. If core elements are missing, a manuscript should be rejected. Revision is necessary when the aspects to be addressed are not adequately described or are not clearly understood.

With the *project report* and *how to* formats, GMS J Med Educ aims to expand the circle of authors and readers beyond the “classic” formats (mainly original and review articles). As the house organ of the DACH Association for Medical Education (GMA), the journal aims to promote exchange among GMA members, but also with all those interested in teaching, learning, and educational research (worldwide), and thus to contribute to the further development of these areas. Since the GMA is a scientific society and a member of the Association of the Scientific Medical Societies in Germany (AWMF), quality standards must also be defined for the article types *project report* and *how to*. Future authors should benefit from the current definition of requirements and criteria, as complying with them is expected to increase the chances of a manuscript being accepted in a transparent and fair review process. In a very pragmatic way, the aspects and rejection criteria we have defined should also facilitate the volunteer work of editors and reviewers by providing a more structured review process. Last but not least, readers should benefit from our proposal, as we aim to improve the overall quality of publications in GMS J Med Educ.

Several journals have stated criteria such as “fills an important gap” [7], or “[filling a] gap in existing practice” [8], “novelty” (e. g. “the idea is new” [7]; “innovations that are novel [...] will be more successful” [8]) or “originality on display” [14] as requirements for a project to be worthy of publication. Kanter (2008) discusses in an editorial that an innovation can be “a creative solution to a problem”, but emphasizes that the difference between the innovation and existing or known solutions must then be described [15]. This meets our requirement to address the innovative nature of a project in the introductory section of a *project report*. However, the discussions among the participants showed that it is a challenge to assess whether a described project is “sufficiently innovative” to justify publication in a scientific journal such as GMS J Med Educ. In introducing the *innovations in medical education* article type, Cook et al. (2010) also addressed the issue of defining “true innovation” and concluded that new approaches to addressing educational and teaching challenges were preferable to existing approaches applied to new topics or new groups of learners [16]. Durning et al. (2020) define *innovation reports* to be published in the Academic Medicine category of the same name as reports of new ideas that have the potential to improve the quality of education and health care by addressing common problems in novel ways, where the approach may be pedagogical, programmatic, or methodological [3]. Durning et al. point out that the category is “deliberately flexible” to cover a wide range of topics, from program evaluations to conceptual contributions. This heterogeneity makes it difficult to assess its innovative character and suggests that decisions need to be made on a case-by-case basis. Accordingly, the authors of the current article concluded that no generalizable rejection criteria can be defined for reviewing *project reports* for innovation.

With regard to the *how to* article type, we believe it is essential that submissions provide a sound rationale for the need for publication and that the content of the manuscript is appropriate for the readership of the GMS J Med Educ. *How to* articles are intended as application-oriented instructions, so a recognizable benefit for practical implementation is considered a prerequisite for publication eligibility. Here, too, scientific standards must be observed, and approaches or procedures must be empirically or theoretically justified. With regard to possible other relevant contexts, the transferability of what has been described is important, but again, no generalizable criteria could be defined for its evaluation.

We describe here the results of a consensus process among the authors of this article. It is obviously difficult to base the results on evidence. However, consideration was given to what experts in the field of teaching, learning, and educational research have published in internationally renowned journals, for example in the form of editorials or commentaries on the formats and questions discussed. In addition, the participants contributed their experiences from different disciplines and professions, reflecting the heterogeneity of publications on training, teaching and learning in the context of different health professions. Finally, the defined criteria must be tested and evaluated in practice.

## Authors’ ORCIDs

- Katrin Schüttpelz-Brauns: [0000-0001-9004-0724]
- Angelika Homberg: [0000-0001-5585-1126]
- Marianne Giesler: [0000-0001-9384-2343]
- Achim Schneider: [0000-0002-8602-8535]
- Pia Gadewoltz: [0009-0000-0632-6536]
- Martin Boeker: [0000-0003-2972-2042]
- Jan Matthes: [0000-0003-2754-1555]

## Acknowledgements

We would like to thank Götz Fabry, editor of GMS J Med Educ, for his invaluable contribution, particularly regarding the core elements. 

## Competing interests

The authors declare that they have no competing interests. 

## Figures and Tables

**Table 1:**
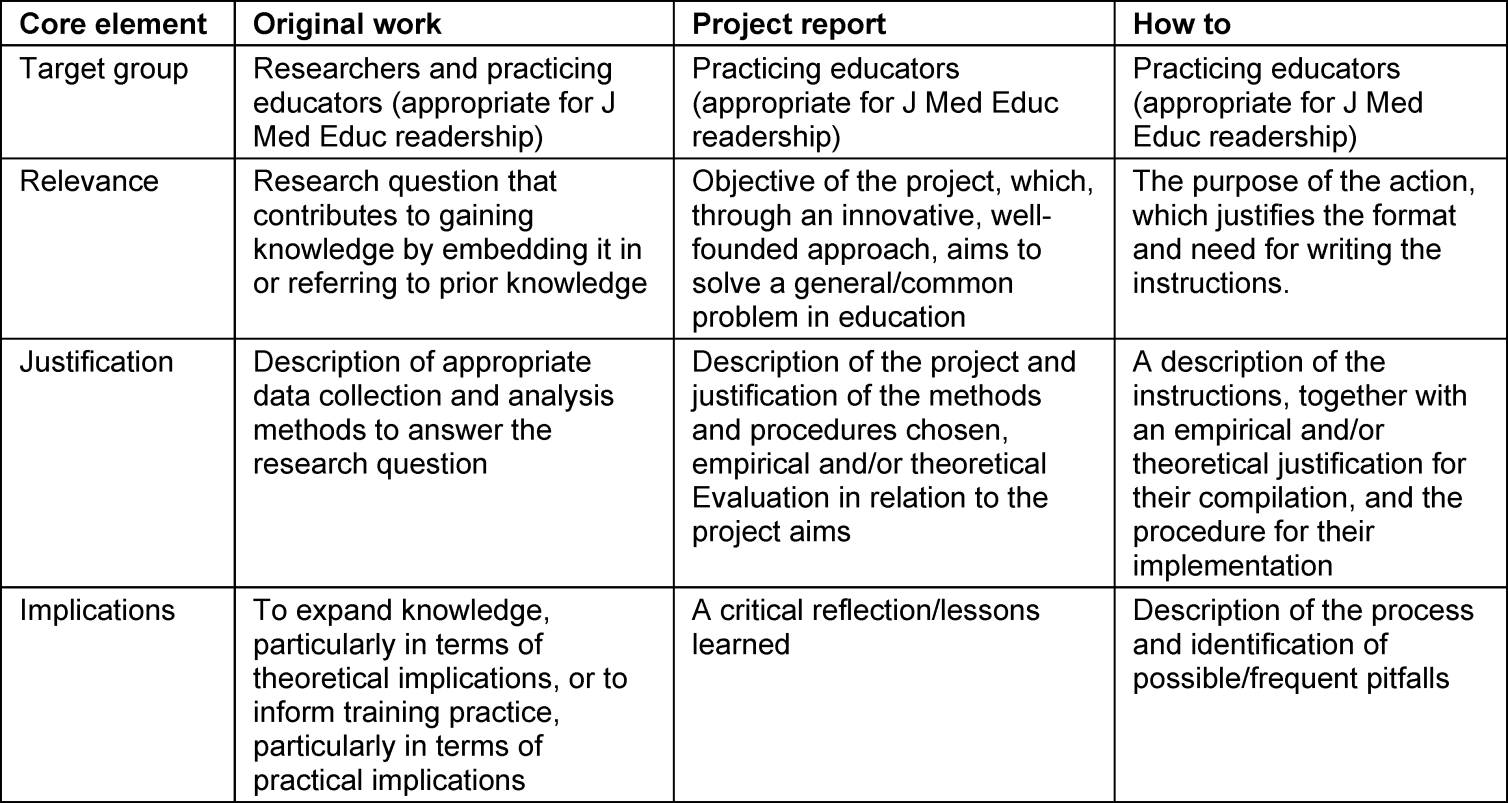
Meaning of the core elements according to article type

**Table 2:**
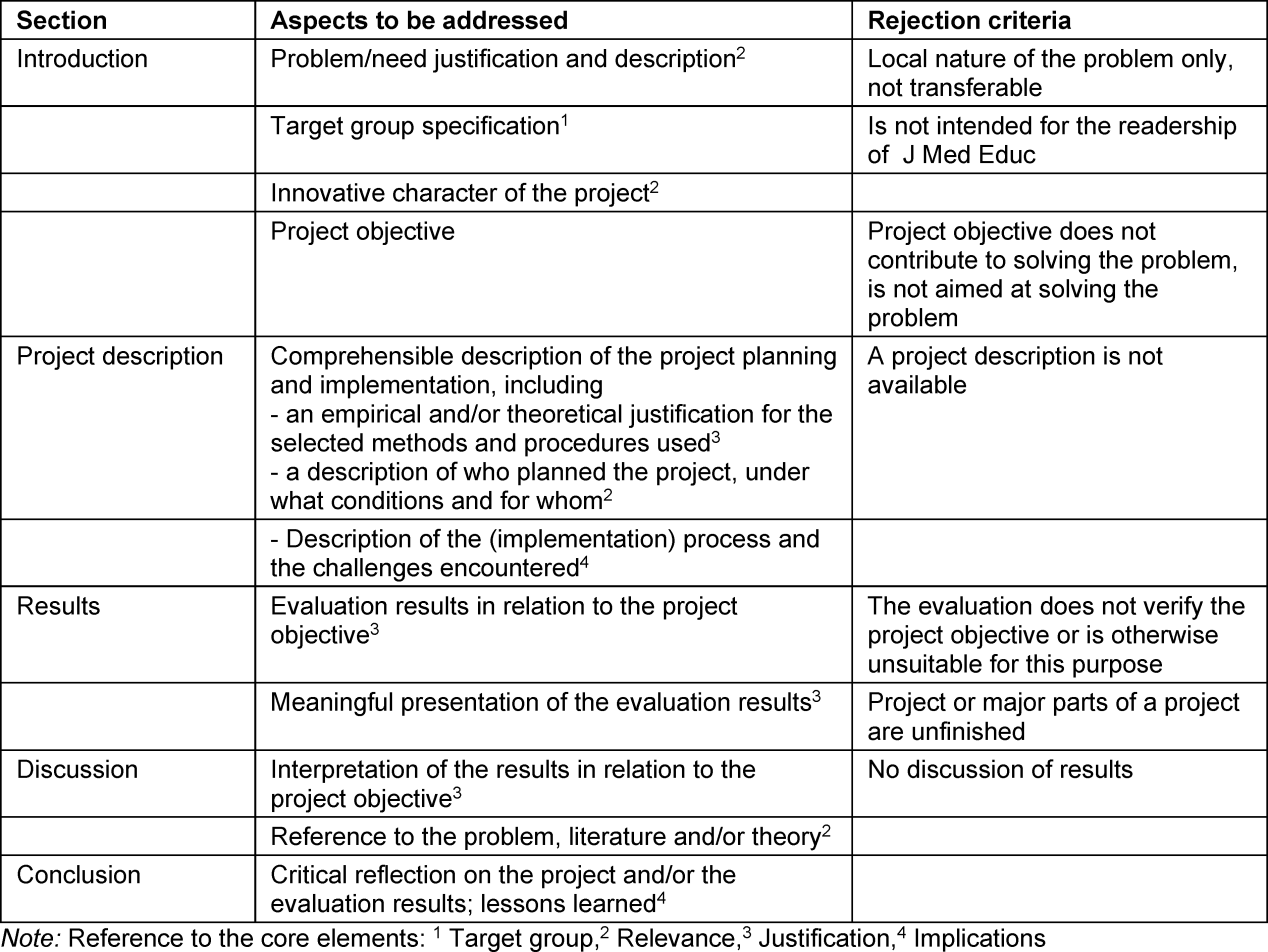
Aspects to be addressed and rejection criteria for the article type project report at GMS J Med Educ

**Table 3:**
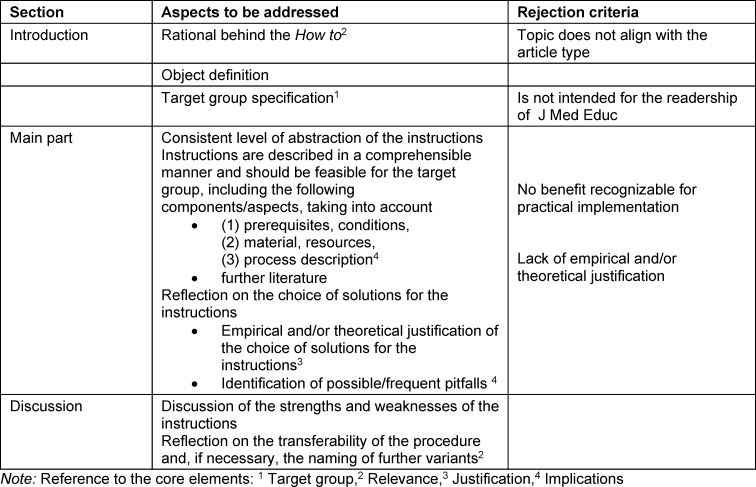
Aspects to be addressed and rejection criteria for the article type how to in GMS J Med Educ
